# Supplementary material for: Elevated CO2 degassing rates prevented the return of Snowball Earth during the Phanerozoic
Source: Nat Commun. 2017 Oct 24;8:1110. doi: 10.1038/s41467-017-01456-w (PMC5736558; doi:10.1038/s41467-017-01456-w)
Supplement: Supplementary file 1 — Supplementary Information [file 41467_2017_1456_MOESM1_ESM.pdf]

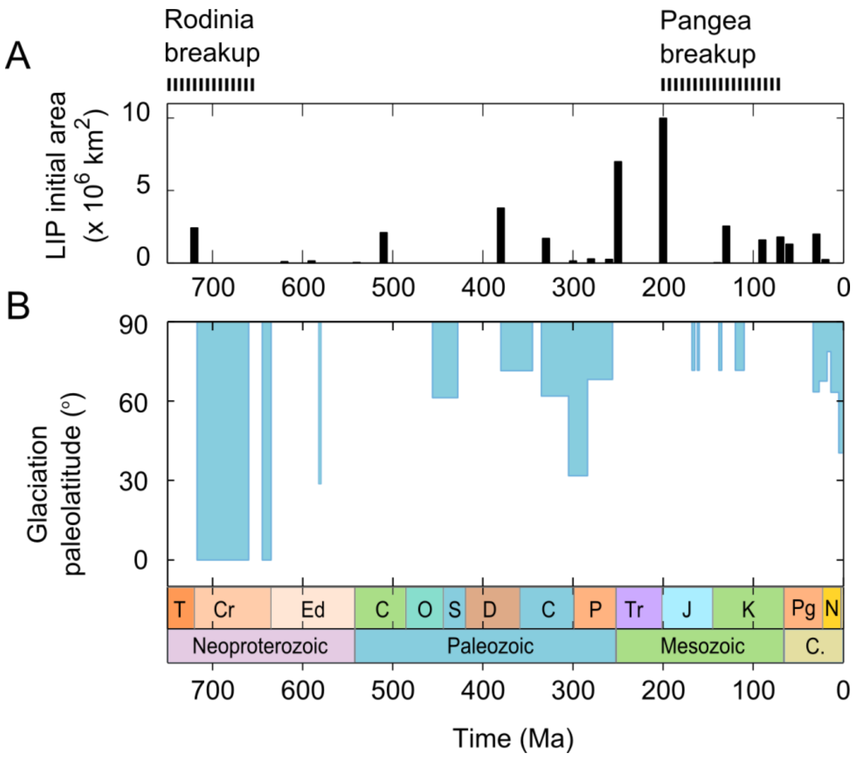

Supplementary Figure 1. ***Glaciations and LIPs.*** *A. Supercontinent breakup and LIP emplacements by initial area[1]. B. Glaciation paleolatitiude record[2].*

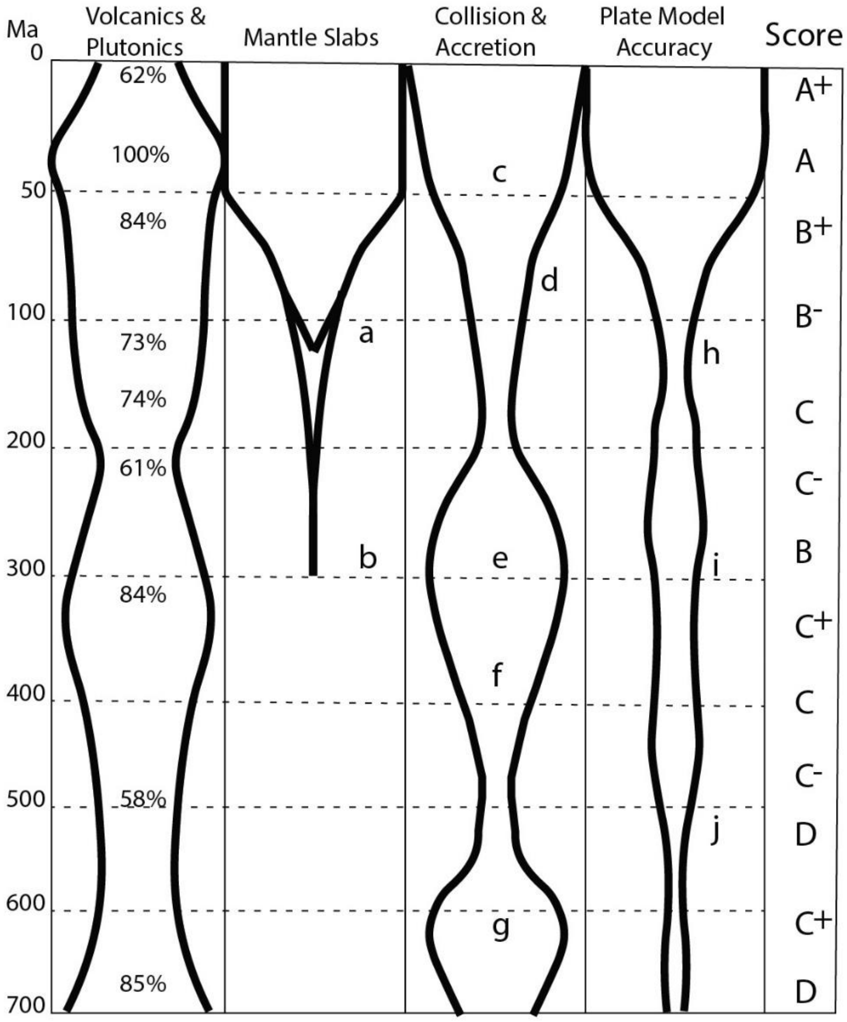

Supplementary Figure 2. ***Evidence Used to Map Ancient Subduction Zones****. A) Subduction-related volcanics, plutonics and accretionary prisms, B) Amoount of subducted material still preserved in the mantle, a- estimate from ref 3, b- estimate from van der Meer et al.*[4]*, C) Times of continental collision and terrane accretion, c- Eurasia, d – Circum-Pacific terranes (e.g. Wrangelia, Omolon), e- Asia terranes (Cimmeria, South China, North China, Amuria), f – Pangea, g – Pannotia, D) Quality of global plate tectonic models, h- oldest ocean floor and hot spots, i - oldest reliable global mean paleomagnetic poles, j-oldest fossils (age constraints).*

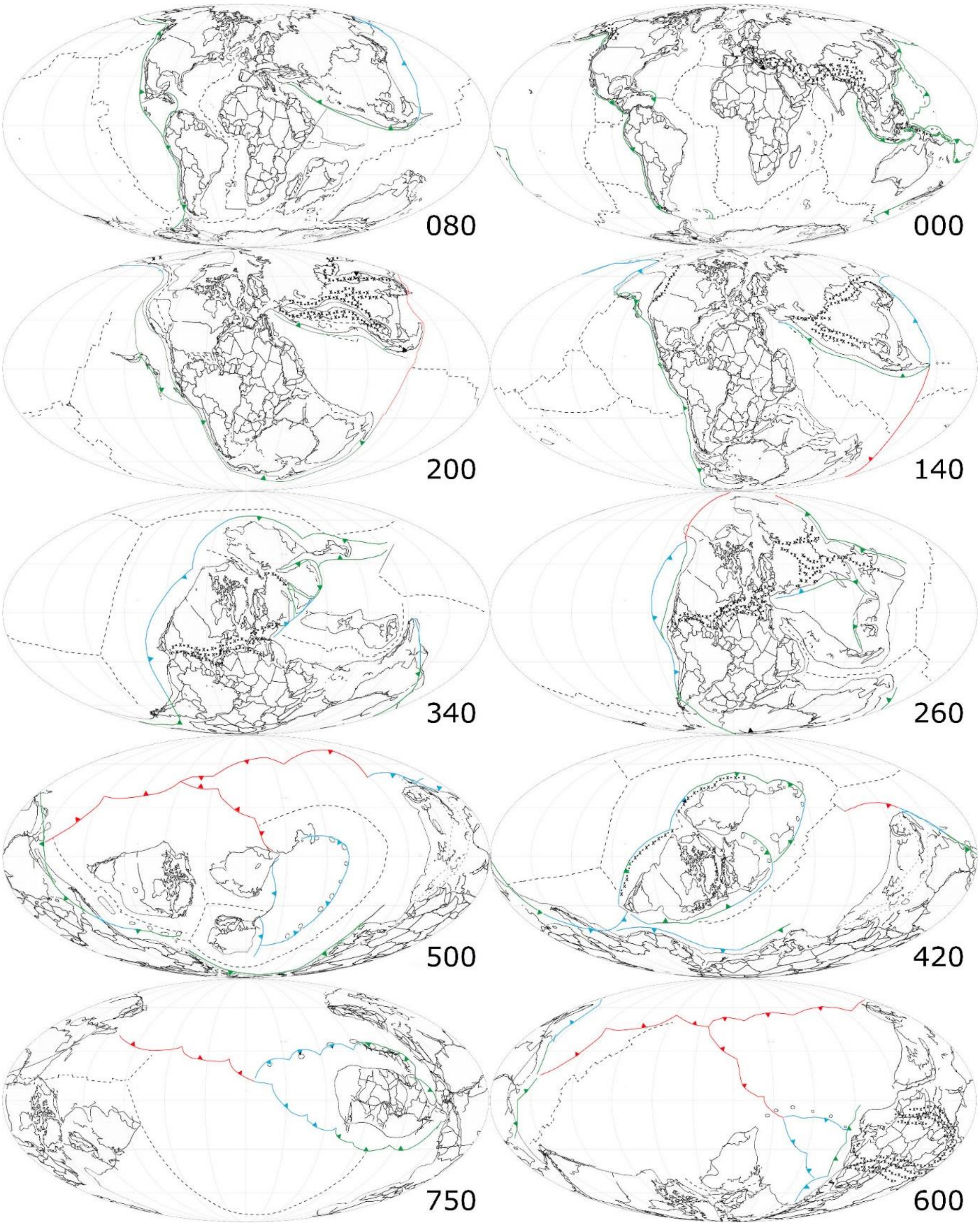

Supplementary Figure 3. ***Selected maps from the PALEOMAP project***. *Green indicates high confidence, blue indicates reasonable confidence and red indicates lower confidence. For further information and full publication list see www.scotese.com.*

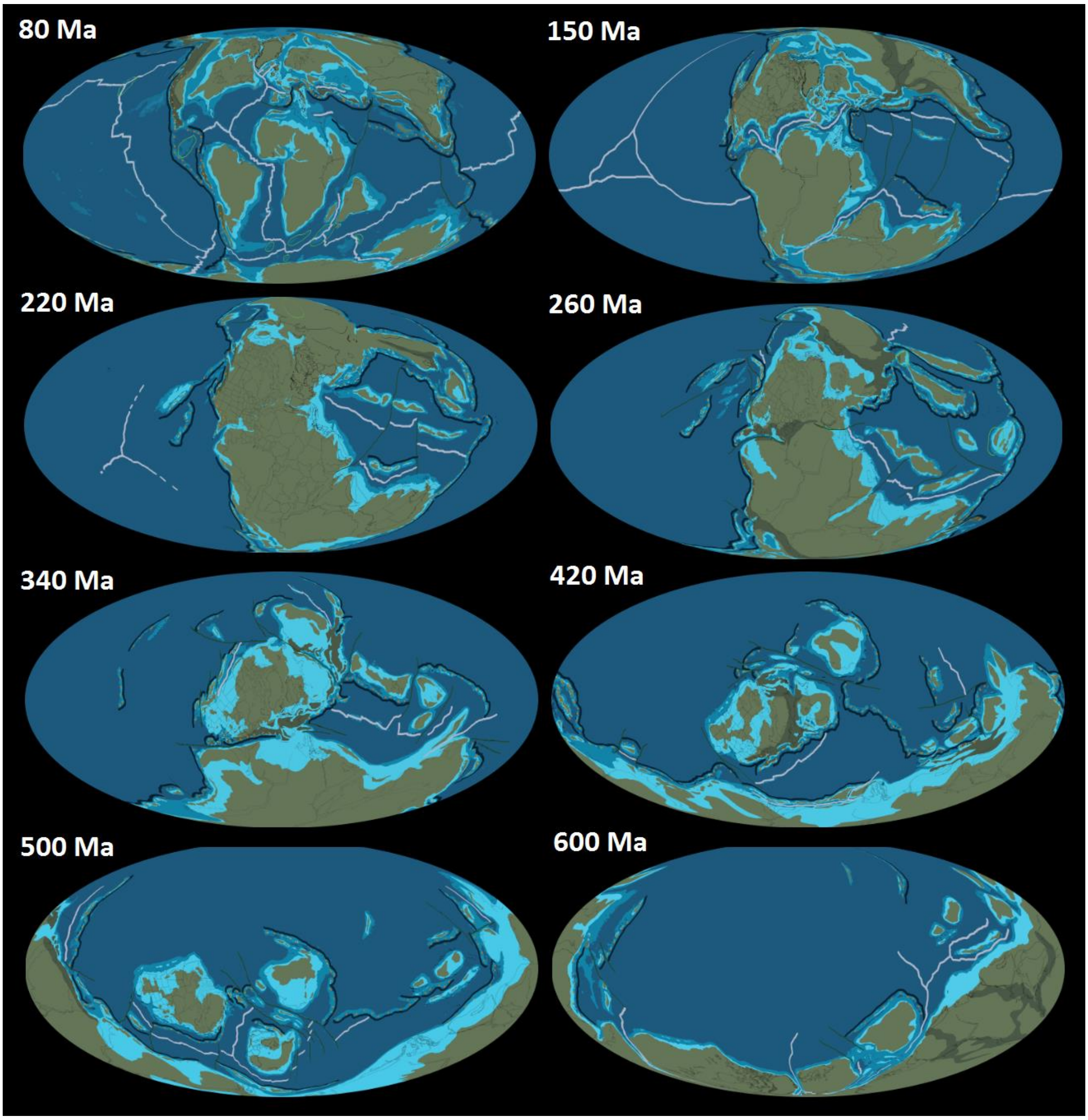

Supplementary Figure 4. ***Selected maps from Deep Time Maps^{TM} for similar periods.*** *Subduction zones are shown in black. For more information and full publication list see* *http://deeptimemaps.com.*

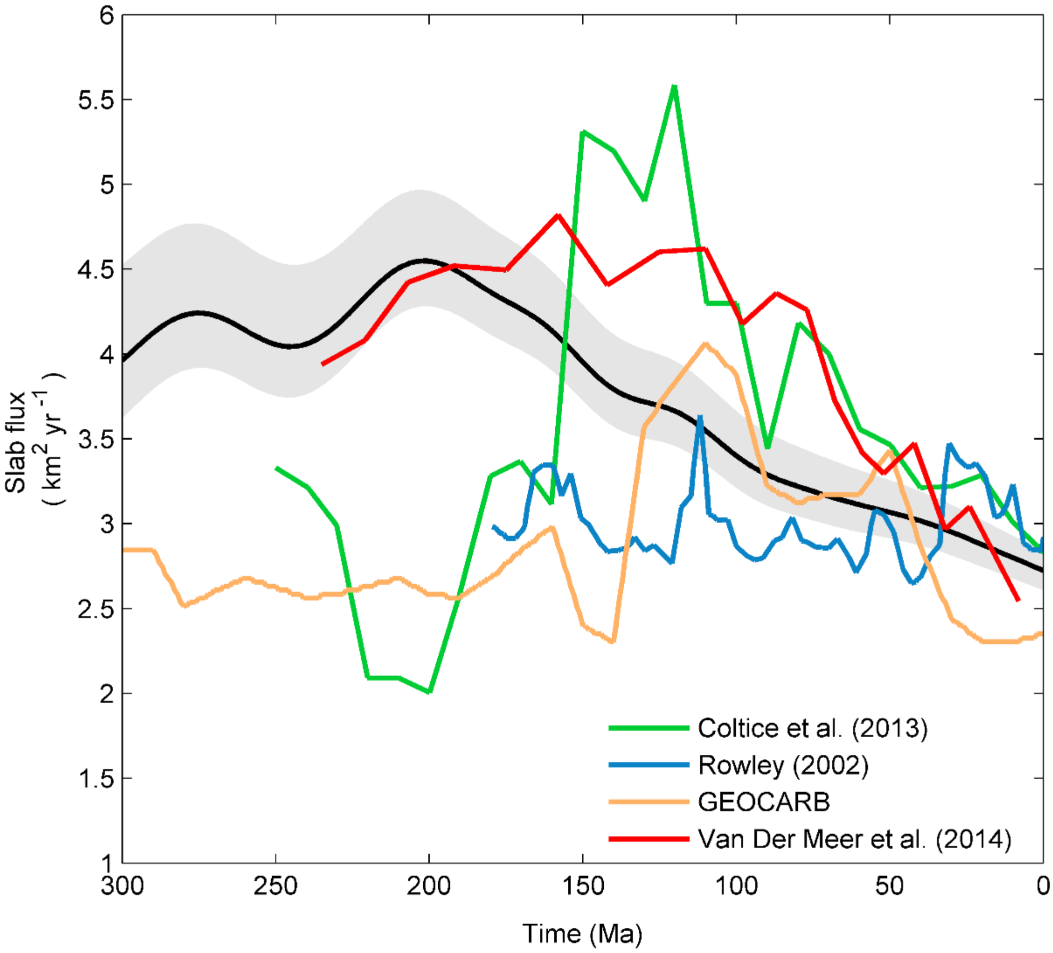

Supplementary Figure 5. ***Global downgoing slab flux.*** *Calculated from the subduction zone length of the PALEOMAP project (black and grey), compared to other studies. 'GEOCARB' shows the curve used in GEOCARBSULF modelling*[5]. Slab flux calculated from our subduction zone length reconstruction (black line), assuming a constant global average subduction rate of 6 cm yr^{-1} (following ref 6). Conservation of crustal mass requires that this slab flux is equal to the total area production at mid ocean ridges, estimations of which are available for the Mesozoic and Cenozoic, based on magnetic anomalies[7-9]. Our reconstruction falls within current estimates, but predicts a sustained high rate for the Triassic and Jurassic, following ref 6.

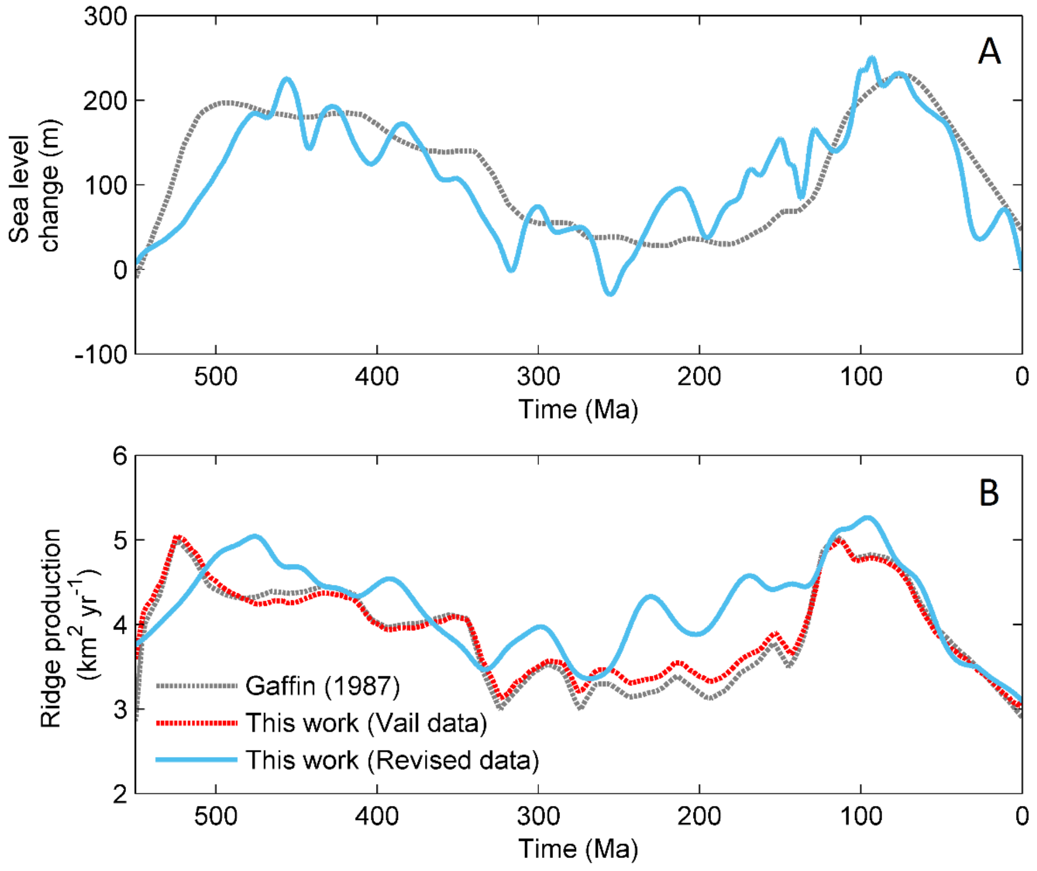

Supplementary Figure 6. Sea-level changes and calculated ridge production rates over the Phanerozoic. Upper panel shows sea-level estimates used in ref 10, which are from the study of Vail et al. (11) (grey line), alongside more recent estimates 12,13 (blue line). Lower panel shows ridge production rates calculated from both datasets, compared to the original production curve of Gaffin[10] (grey line).

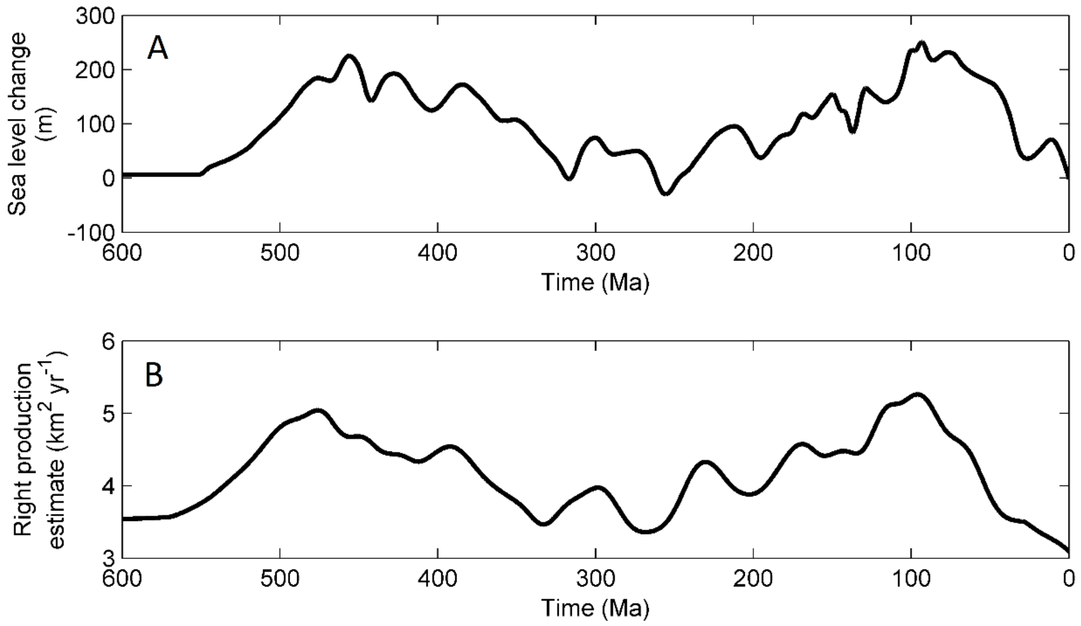

Supplementary Figure 7. Sea-level changes and calculated ridge production rates over the Late Neoproterozoic and Phanerozoic. As S6 but with the assumption that sea level from 600 Ma – 550 Ma is fixed at the value for 520 Ma.

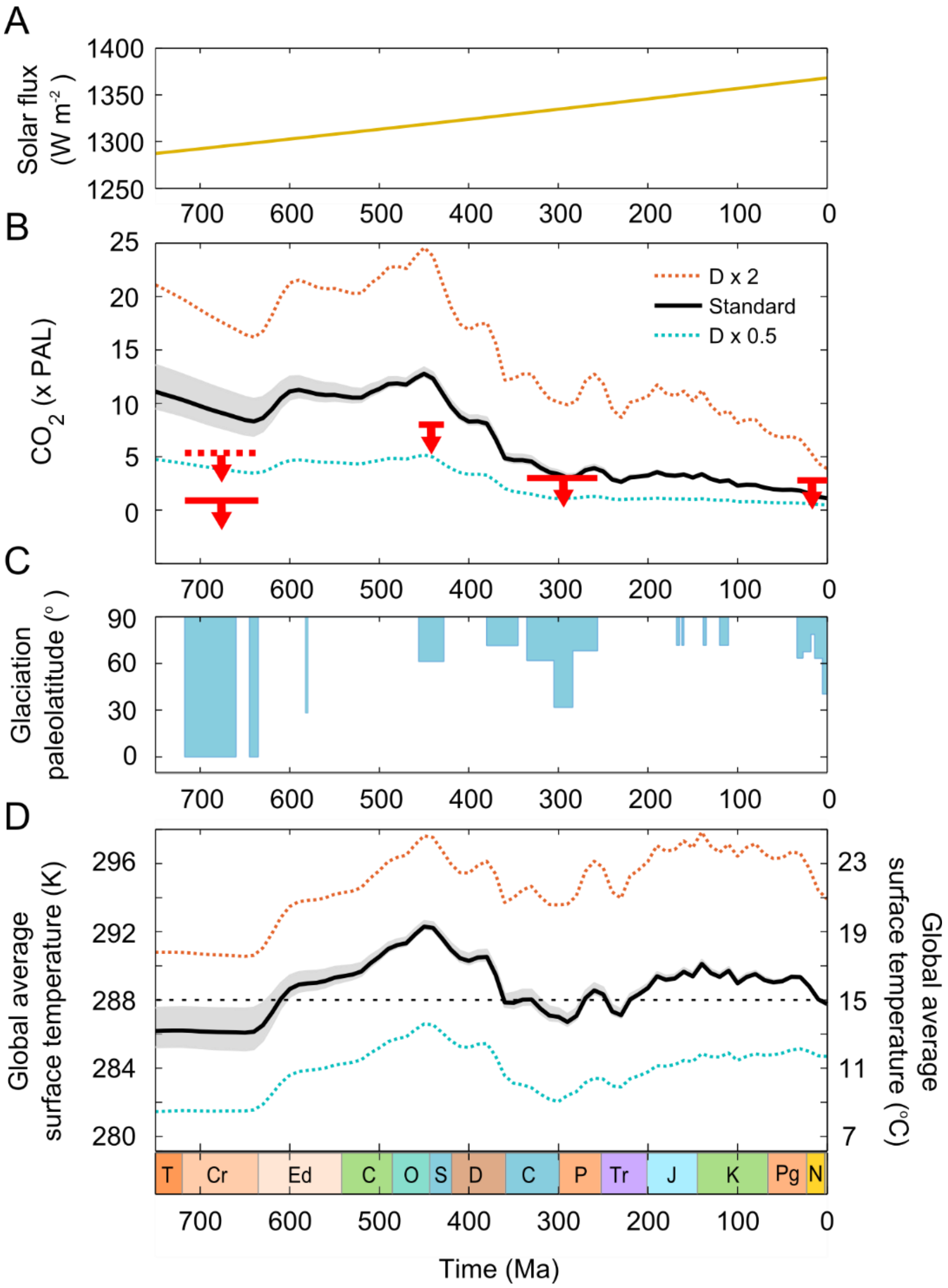

Supplementary Figure 8. Biogeochemical model predictions for varying the degassing rate. A. Model solar flux. B: relative atmospheric CO_2 concentration shown against suggested thresholds for glaciation (red bars, see ms). C: Glaciation paleolatitude, as in Fig 1. D: modelled global average surface temperature. For model runs, black lines show standard run as in COPSE model, with PALEOMAP degassing (see ms), blue lines show PALEOMAP degassing divided by two, and orange lines double the degassing rate. Oscillations between greenhouse and icehouse conditions, including the severe glaciations of the Cryogenian, may

potentially be triggered by variation in degassing rate alone, without the requirement for a 'weathering enhancement trigger'. Variations in degassing rate may be driven by changes in plate velocity, the proportion of continental arc volcanism (i.e. liberating additional crustal carbon), and the overall carbon content of the crust. It is unlikely that any of these factors underwent a significant decrease during the Late Neoproterozoic-Paleozoic transition[14,15], therefore our conclusion of a rising global CO_2 input rate over this time appears robust to such sensitivity.

Supplementary Note 1.

Full model equations.

Full equations are documented below. Almost all follow the original COPSE model[16] others were altered in subsequent work[17]. RO_2 and RCO_2 denote concentrations of oxygen and carbon dioxide relative to present day. Subscript zeros represent the present day size of fluxes and reservoirs. The only change made for this paper is the revision of the seafloor weathering term[18], and the imposition of the new degassing rate forcing (D). Fluxes are listed below.

Marine organic carbon burial: $$mocb = k_{mocb}\left(\frac{P}{P_0}\right)^2 \quad (1)$$

Phosphorus weathering: $$phosw = k_{phosw}\left(\frac{2}{12}\frac{silw}{silw_0} + \frac{5}{12}\frac{carbw}{carbw_0} + \frac{5}{12}\frac{oxidw}{oxidw_0}\right) \quad (2)$$

Marine organic phosphorus burial: $$mopb = \frac{mocb}{CPsea} \quad (3)$$

Calcium-bound phosphorus burial: $$capb = k_{capb}\frac{mocb}{mocb_0} \quad (4)$$

Iron-sorbed phosphorus burial: $$fepb = k_{fepb}\cdot\frac{(1-anox)}{k_{oxfrac}} \quad (5)$$

Silicate weathering: $silw = k_{silw} \cdot W \cdot U \cdot f_T \cdot \sqrt{RCO_2}$ (6)

Carbonate weathering: $carbw = k_{carbw} \cdot W \cdot U \cdot g_T \cdot \sqrt{RCO_2}$ (7)

Oxidative weathering: $oxidw = k_{oxidw} \cdot U \cdot \sqrt{RO_2}$ (8)

Marine carbonate carbon burial: $mccb = silw + carbw$ (9)

Seafloor weathering: $sfw = k_{sfw} \cdot D \cdot e^{0.061(T-T_0)}$ (10)

Pyrite sulphur weathering: $pyrw = k_{pyrw} \cdot U \cdot \frac{PYR}{PYR_0}\sqrt{RO_2}$ (11)

Gypsum sulphur weathering: $gypw = k_{gypw} \cdot U \cdot \frac{GYP}{GYP_0} \cdot \frac{carbw}{carbw_0}$ (12)

Pyrite sulphur burial: $mpsb = k_{mpsb} \cdot \frac{S}{S_0} \cdot \frac{1}{O} \cdot \frac{mocb}{mocb_0}$ (13)

Gypsum sulphur burial: $mgsb = k_{mgsb} \cdot \frac{S}{S_0}$ (14)

Pyrite sulphur degassing: $pyrdeg = k_{pyrdeg\left(\frac{PYR}{PYR_0}\right)\frac{dA}{dt}}$ (15)

Gypsum sulphur degassing: $gypdeg = k_{gypdeg\left(\frac{GYP}{GYP_0}\right)\frac{dA}{dt}}$ (16)

Organic carbon degassing: $ocdeg = k_{ocdeg\left(\frac{G}{G_0}\right)\frac{dA}{dt}}$ (17)

Carbonate carbon degassing: $ccdeg = k_{ccdeg\left(\frac{C}{C_0}\right)\frac{dA}{dt}}$ (18)

Model variable calculations are included below.

Relative atmospheric CO_2*: $RCO_2 = e^{k_a(T_0-T)}\left(\frac{\sum CO_2}{(\sum CO_2)_0}\right)^2$ (19)

where $\sum CO_2$ is total oceanic carbon

Relative atmospheric O_2: $RO_2 = \frac{\frac{O}{O_0}}{\frac{O}{O_0}+k_{O2}}$ (20)

where $k_{O2} = 3.762$

Solar forcing: $S = \frac{S_0}{1+0.38\left(\frac{t}{\tau}\right)}$ (21)

where $S_0 = 1368 Wm^{-2}$, $\tau = 4.55x10^9$ years.

Following COPSE, the global average surface temperature calculation is taken from the model of Caldeira and Kasting[19] and requires inputs of solar forcing, albedo and carbon dioxide concentration. This follows the COPSE treatment.

Model reservoir calculations are included below.

Ocean phosphate: $\frac{dP}{dt} = phosw - mopb - capb - fepb$ (22)

Hydrosphere carbon: $\frac{dA}{dt} = carbw + ccdeg + oxidw + ocdeg - mccb - mocb - sfw$ (23)

Ocean sulphate: $\frac{dS}{dt} = pyrw + pyrdeg + gypw + gypdeg - mpsb - mgsb$ (24)

Buried organic C: $\frac{dG}{dt} = mocb - oxidw - ocdeg$ (25)

Buried carbonate C: $\frac{dC}{dt} = mccb + sfw - carbw - ccdeg$ (26)

Buried pyrite S: $\frac{dPYR}{dt} = mpsb - pyrw - pyrdeg$ (27)

Buried Gypsum S: $\frac{dPYR}{dt} = mgsb - gypw - gypdeg$ (28)

**Present day values:**			**Source:**
Marine organic carbon burial:	$k_{mocb}=4.5x10^{12}$	mol C yr^{-1}	COPSE
Calcium-bound P burial:	$k_{capb}=1.5x10^{10}$	mol P yr^{-1}	COPSE
Pyrite sulphur burial:	$k_{mpsb}=7x10^{11}$	mol S yr^{-1}	for steady state

Gypsum sulphur burial:	k_{mgsb}=2.5x10^{12}	mol S yr^{-1}	for steady state
Silicate weathering:	k_{silw} = 4.9x10^{12}	mol C yr^{-1}	for steady state
Seafloor weathering:	k_{sfw} = 1.75x10^{12}	mol C yr^{-1}	ref. 17
Oxidative weathering:	k_{oxidw}=7.75x10^{12}	mol C yr^{-1}	for steady state
Reactive P weathering:	k_{phosw}=4.9x10^{10}	mol P yr^{-1}	for steady state
Pyrite sulphur weathering:	k_{pyrw}=4.5x10^{11}	mol S yr^{-1}	GEOCARBSULF
Gypsum sulphur weathering:	k_{gypw}=2x10^{12}	mol S yr^{-1}	GEOCARBSULF
Organic carbon degassing:	k_{ocdeg}=1.25x10^{12}	mol C yr^{-1}	COPSE
Carbonate carbon degassing:	k_{ccdeg}=6.65x10^{12}	mol C yr^{-1}	COPSE
Pyrite sulphur degassing:	k_{pyrdeg}= 2.5x10^{11}	mol S yr^{-1}	GEOCARBSULF
Gypsum sulphur degassing:	k_{gypdeg} = 5.x10^{11}	mol S yr^{-1}	GEOCARBSULF
Atmosphere and ocean CO_2:	A_0=3.193x10^{18}	mol	COPSE
Ocean phosphate:	P_0=3.1x10^{15}	mol	COPSE
Ocean sulphate:	P_0=4x10^{19}	mol	COPSE
Atmosphere and ocean oxygen:	O_0=3.7x10^{19}	mol	COPSE
Buried organic carbon:	G_0=1.9x10^{21}	mol	ref. 17
Buried carbonate carbon:	C_0=6.6x10^{21}	mol	ref. 17
Buried pyrite sulphur:	PYR_0=1.8x10^{20}	mol	COPSE
Buried gypsum sulphur:	GYP_0=2x10^{20}	mol	COPSE
Carbon-to-phosphorus burial ratio (CPsea):	250	mol/mol	COPSE

Model forcings follow the below description.

Relative degassing rate:	$D = 1$ for present day,<br>PALEOMAP forcing
Relative uplift rate:	$U = 1$ for present day<br>fixed at present day value for Precambrian,<br>COPSE forcing for Phanerozoic
Terrestrial weatherability:	W = 1 for present day,<br>$W \approx \frac{1}{4}$ before vascular plants (ref 5)

Supplementary References

1. Ernst, R. E. *Large igneous provinces.* Cambridge University Press. Cambridge, United Kingdom (2014).

2. Cather, S. M., Dunbar, N. W., McDowell, F. W., McIntosh, W. C. & Scholle, P. A. Climate forcing by iron fertilization from repeated ignimbrite eruptions: The icehouse-silicic large igneous province (SLIP) hypothesis. *Geosphere* **5**, 315-324, doi:10.1130/ges00188.1 (2009).

3. Domeier, M., Doubrovine, P. V., Torsvik, T. H., Spakman, W. & Bull, A. L. Global correlation of lower mantle structure and past subduction. *Geophysical Research Letters* **43**, 4945-4953, doi:10.1002/2016gl068827 (2016).

4. Van Der Meer, D.G., Spakman, W., van Hinsbergen, D.J.J., Amaru, M.L., and Torsvil, T. Towards absolute plate motions constrained by lower-mantle slab remanents, *Nature Geoscience* **3,** 36-40 (2010).

5. Berner, R. A. GEOCARBSULF: A combined model for Phanerozoic atmospheric O_2 and CO_2. *Geochimica et Cosmochimica Acta* 70, 5653-5664 (2006).

6. Van Der Meer, D. G. *et al.* Plate tectonic controls on atmospheric CO2 levels since the Triassic. *PNAS* **111**, 4380-4385 (2014).

7. Engbretson, D. C., Kelley, K. P., Cashman, H. J. & Richards, M. A. 180 Million years of subduction. *GSA Today* 2, 93-100 (1992).

8. Rowley, D. B. Rate of plate creation and destruction: 180 Ma to present. *Geological Society of America Bulletin* 114, 927-933 (2002).

9. Coltice, N., Seton, M., Rolf, T., Müller, R. D. & Tackley, P. J. Convergence of tectonic reconstructions and mantle convection models for significant fluctuations in seafloor spreading. *Earth and Planetary Science Letters* 383, 92-100, doi:10.1016/j.epsl.2013.09.032 (2013).

10. Gaffin, S. Ridge volume dependence on seafloor generation rate and inversion using long term sealevel change. *American Journal of Science* **287**, 596-611 (1987).

11. Vail, P. R., Mitchum, R. M. and Thompson, S. Seismic stratigraphy and global changes of sea level: *American Association of Petroleum Geologists Memoirs* **36,** 129-144 (1977).

12. Haq, B. U. Cretaceous eustasy revisited. *Global and Planetary Change* **113**, 44-58, doi:10.1016/j.gloplacha.2013.12.007 (2014).

13. Snedden, J. and Liu, C. A compilation of Phanerozoic sea-level change, coastal onlaps and recommended sequence designations. *American Association of Petroleum Geologists Search and Discovery Article* 40594 (2010).

14. Hayes, J. M. & Waldbauer, J. R. The carbon cycle and associated redox processes through time. *Phil. Trans. R. Soc. B* **361**, 931-950 (2006).

15. McKenzie, N. R., Horton, B. K., Loomis, S. E., Stockli, D. F., Planavsky, N. J. & Cin-Ty, A. L. Continental arc volcanism as the principal driver of icehouse-greenhouse variability. *Science* 352, 444-447 (2016).

16. Bergman, N. M., Lenton, T. M. & Watson, A. J. COPSE: A new model of biogeochemical cycling over Phanerozoic time. *American Journal of Science* **304**, 397-437 (2004).

17. Mills, B., Lenton, T. M. & Watson, A. J. Proterozoic oxygen rise linked to shifting balance between seafloor and terrestrial weathering. *Proceedings of the National Academy of Sciences of the United States of America* 111, 9073-9078,

18. Mills, B. J. W., Belcher, C. M., Lenton, T. M. & Newton, R. J. A modeling case for high atmospheric oxygen concentrations during the Mesozoic and Cenozoic. *Geology* **44**, 1023-1026, doi:10.1130/g38231.1 (2016).

19. Caldeira, K. & Kasting, J. F. The life span of the biosphere revisited. *Nature* **360**, 721-723 (1992).
